# Associations Between Physical Activity and Alcohol Consumption in Rural Cancer Survivors

**DOI:** 10.3389/fonc.2022.871192

**Published:** 2022-06-07

**Authors:** Scherezade K. Mama, Natalia I. Heredia, Hannah Johnston, David E. Conroy

**Affiliations:** ^1^ Department of Health Disparities Research, Division of Cancer Prevention and Population Sciences, The University of Texas MD Anderson Cancer Center, Houston, TX, United States; ^2^ Department of Health Promotion and Behavioral Sciences, School of Public Health, The University of Texas Health Science Center at Houston, Houston, TX, United States; ^3^ Department of Behavioral Science, Division of Cancer Prevention and Population Sciences, The University of Texas MD Anderson Cancer Center, Houston, TX, United States; ^4^ Department of Kinesiology, College of Health and Human Development, The Pennsylvania State University, University Park, PA, United States

**Keywords:** physical activity, alcohol use, cancer survivorship, rural health, health disparities

## Abstract

**Purpose:**

Rural adults and cancer survivors are more likely to be physically inactive and exceed recommendations for alcohol use. Physical activity and alcohol use are positively associated in adults and cancer survivors but associations between physical activity and alcohol use in rural cancer survivors is unknown. This cross-sectional study explored associations between physical activity, sitting time, and alcohol use in rural cancer survivors.

**Methods:**

Cancer survivors residing in central Pennsylvania were recruited to the Partnering to Prevent and Control Cancer (PPCC) study and completed mailed questionnaires assessing physical activity (low, moderate, high), sitting time (<6 or ≥6 hours/day), and alcohol use (0 or ≥1 drinks/week). Binary logistic regression models tested associations between physical activity, sitting time, and alcohol use, adjusting for age, gender, and education.

**Results:**

Participants (N=219) were in their mid-60s (M age=64.5 ± 12.2 years, 60.7% female), overweight (M BMI=29.6 ± 6.9 kg/m2), and 50.5% were college graduates. Nearly half of participants were breast (22.8%) or prostate (20.5%) cancer survivors and 90.4% were >12 weeks but <5 years post-treatment. Participants self-reported meeting physical activity recommendations (79.5%), sitting <6 hours/day (53.3%), and consuming ≥1 alcoholic drinks/week (54.1%). Participants who reported being moderately (OR=5.0, 95% CI: 1.9-12.9) or highly (OR=4.5, 95% CI: 1.9-10.9) active had higher odds of reporting alcohol use, after adjusting for covariates.

**Conclusion:**

Results mirror positive associations seen in adults and other subgroups (e.g., racial/ethnic minority adults). Cancer control efforts should stress being physically active while emphasizing messaging to curtail increases in alcohol use among rural cancer survivors.

## Introduction

Physical inactivity and alcohol use are common behaviors associated with increased cancer risk (1–5). Physical activity recommendations for adults are 150 minutes of moderate intensity or greater aerobic activity and muscle-strengthening activities at least two days per week (6). Recommendations for alcohol use in adults are to not drink at all or to drink in moderation by limiting intake to two drinks or less per day for men; and 1 drink or less per day for women (7). Although health risk behaviors are expected to cluster in a functionally consistent manner (8), physical activity has consistently been associated with greater alcohol use (9–11). However, we are not aware of any studies that examined this association in rural cancer survivors. Adults residing in rural areas tend to be older, poorer, and sicker (12) and engage in more health risk behaviors than their urban counterparts (13). In addition to being less active and more sedentary (14, 15), rural adults tend toward extremes of alcohol use compared to their urban counterparts (16). Although more likely to abstain from alcohol use, rural adults who drink alcohol are more likely to be moderate or heavy drinkers (13). Furthermore, over half of cancer survivors in the United States identify as drinkers (17, 18).

To our knowledge, only one study has examined associations between physical activity and alcohol use in cancer survivors and found that survivors who engaged in greater light intensity physical activity were more likely to consume alcohol (19). That study focused on urban breast cancer survivor, and it is unclear whether findings would generalize to rural cancer survivors or hold true for sedentary behavior. Thus, we explored associations between physical activity, sitting time, and alcohol use in a sample of rural cancer survivors and hypothesized that physical activity would be positively associated with alcohol use in this secondary analysis.

## Methods

Rural cancer survivors were recruited to a cross-sectional study to understand barriers and preferences for physical activity adoption and maintenance. The study was approved by the Institutional Review Boards at The Pennsylvania State University and The University of Texas MD Anderson Cancer Center and informed consent was provided prior to participation. Detailed descriptions of recruitment methods, participants, and procedures have been published previously and are described briefly below (20, 21).

Eligible adults had a history of cancer, lived within a 28-county area in central Pennsylvania, and were English-speaking. Participants were mailed a brief questionnaire assessing demographics (e.g., age, gender, education) and cancer history (e.g., type, time since diagnosis, treatment status). Participants who returned the brief questionnaire were enrolled in the study and sent additional questionnaires assessing physical activity, sitting time, and alcohol use. The International Physical Activity Questionnaire (IPAQ) long form was used to assess physical activity and sitting time (22, 23). The IPAQ assesses domain-specific (e.g., occupation, leisure-time) and intensity-based (e.g., moderate, vigorous) physical activity. Participants reported the frequency and duration for each activity over the last 7 days and the average duration of sitting time on weekends and weekdays. To assess alcohol use over the past three months, participants were asked to report the number of drinks consumed on average for each day of the week, the maximum number of drinks they consumed on any one occasion, and the number of times they consumed five or more drinks over the past three months (24). A drink was defined as one beer, one wine cooler, one glass of wine, one shot, or one mixed drink.

Physical activity, sitting time, and alcohol use were categorized as shown in **Table 1** and binary logistic regression models were used to explore associations between physical activity, sitting time, and alcohol use, adjusting for age, gender, and education. Statistical analyses were performed using SPSS version 24 (IBM SPSS Inc., Armonk, NY). All statistical tests were 2-sided, and *p* less than .05 was considered statistically significant.

**Table 1:** Participant characteristics.

Characteristic	*N* (Percent)
Gender	
Female	133 (60.7)
Male	86 (39.3)
Age [mean ± *SD* years]	64.5 ± 12.2
BMI [mean ± *SD* kg/m^2^]	29.6 ± 6.9
Weight status	
Underweight/normal weight	66 (30.6)
Overweight	62 (28.7)
Obese	88 (40.7)
Education	
< Bachelor’s degree	108 (49.5)
Bachelor’s degree	56 (25.7)
> Bachelor’s degree	54 (24.8)
Annual household income	
< $40,000	40 (19.4)
$40,000-79,999	66 (30.1)
≥ $80,000	100 (48.5)
Cancer type	
Breast	50 (22.8)
Colorectal	20 (9.1)
Gynecological	37 (16.9)
Prostate	45 (20.5)
Other	67 (30.6)
Treatment status	
Currently undergoing treatment	21 (9.7)
≥ 12 weeks post-surgery or treatment	169 (90.4)
> 5 years post-surgery or treatment	18 (9.6)
Self-rated health	
Poor or fair	35 (16.1)
Good, very good, or excellent	182 (83.8)
Physical activity^a^	
Low	45 (20.5)
Moderate	65 (29.7)
High	109 (49.8)
Sitting time	
< 6 hours/day	112 (53.3)
≥ 6 hours/day	98 (46.7)
Alcohol use	
Does not drink (0 drinks/week)	95 (45.9)
Drinks alcohol (≥ 1 drinks/week)	112 (54.1)
Binge drinking	
Does not binge drink (< 5 drinks/time)	180 (87.4)
Binge drinks (≥ 5 drinks/time)	26 (12.6)

a Low, moderate, and high physical activity categories refer to individuals who do not meet recommendations, meet recommendations of 150 minutes of moderate-intensity physical activity each week, and exceed recommendations, respectively.

## Results

Of the 263 rural cancer survivors enrolled in PPCC, 219 (83.3%) completed all questionnaires. Participant characteristics, physical activity, sitting time, and alcohol use are shown in **Table 1**. Participants were mostly women, in their mid-60s with overweight or obesity and reported high socioeconomic status. Nearly half of the participants were post-treatment breast or prostate cancer survivors.

Most cancer survivors self-reported meeting (29.7%) or exceeding (49.8%) physical activity recommendations of 150 minutes of moderate or greater-intensity physical activity each week (6, 25). Compared to those who reported low (24.4%) physical activity and did not meet recommendations, more cancer survivors who reported moderate (58.7%) or high (63.1%) physical activity drank alcohol at least once per week [χ^2^(2)=18.5, *p*<.001]. Physical activity was not significantly associated with binge drinking [χ^2^(2)=5.1, *p*=.078], and sitting time was not associated with either alcohol use [χ^2^(1)=2.2, *p*=.138] or binge drinking [χ^2^(1)=0.9, *p*=.334].

Associations between physical activity, sitting time, and alcohol use are shown in **Table 2**. Cancer survivors who reported moderate (OR=4.4, 95% CI: 1.8-10.5) or high (OR=5.3, 95% CI: 2.3-12.0) physical activity had over four times the odds of consuming alcohol at least once per week than those who reported low activity in unadjusted models. After adjusting for age, gender, and education, cancer survivors who reported moderate (OR=5.0, 95% CI: 1.9-12.9) or high (OR=4.5, 95% CI: 1.9-10.9) physical activity had greater odds of consuming alcohol than those who reported low physical activity. There were no significant associations between physical activity and binge drinking or sitting time and alcohol use in unadjusted or adjusted models.

**Table 2:** Associations between physical activity and sitting time and alcohol use in rural cancer survivors [OR (95% CI)].

Model	Alcohol Use		Binge Drinking	
	Unadjusted	Adjusted^a^	Unadjusted	Adjusted^a^
Physical Activity				
Low	ref	ref	ref	ref
Moderate	**4.412 (1.845-10.547)**	**5.015 (1.949-12.907)**	2.053 (0.394-10.702)	1.727 (0.302-9.895)
High	**5.303 (2.341-12.009)**	**4.522 (1.881-10.867)**	4.179 (0.924-18.904)	2.596 (0.528-12.771)
Age		0.974 (0.948-1.000)		**0.916 (0.874-0.960)**
Gender				
Female		ref		ref
Male		**3.702 (1.861-7.363)**		**11.594 (3.539-37.985)**
Education				
> Bachelor’s degree		ref		ref
Bachelor’s degree		**0.413 (0.193-0.884)**		1.506 (0.467-4.853)
< Bachelor’s degree		0.664 (0.275-1.607)		0.976 (0.253-3.770)
Sitting Time				
< 6 hours/day	ref	ref	ref	ref
≥ 6 hours/day	0.655 (0.374-1.146)	0.634 (0.346-1.161)	0.650 (0.270-1.564)	0.613 (0.230-1.634)
Age		**0.970 (0.945-0.995)**		**0.916 (0.874-0.959)**
Gender				
Female		ref		ref
Male		**3.279 (1.663-6.352)**		**12.673 (3.702-43.386)**
Education				
> Bachelor’s degree		ref		ref
Bachelor’s degree		**0.395 (0.187-0.835)**		1.875 (0.532-6.606)
< Bachelor’s degree		0.621 (0.261-1.476)		1.357 (0.325-5.677)

a Models adjusted for age, gender and education.

Bold face indicates odds ratios that are statistically significant (p<.05).

## Discussion

Physical activity and alcohol use were positively associated in rural cancer survivors in this study, mirroring results for adults (9–11) and other subgroups, including African American adults, Mexican American adults, and cancer survivors (19, 26, 27). Understanding the co-occurrence of behavioral risk factors is critical to developing effective behavioral interventions to reduce cancer risk and recurrence (28–30). Our results emphasize the need for multiple health behavior change interventions that stress the importance of being physically active while curtailing alcohol use among rural cancer survivors.

Although there has been recent emphasis on the link between alcohol and cancer, the cancer burden due to alcohol is not fully understood (31–33) and less is known about how alcohol impacts cancer survivorship, long-term outcomes, and cancer health disparities (34). Our findings work toward filling that gap for rural cancer survivors who tend to be less physically active and report greater alcohol consumption (14, 16). Limitations of this study include the cross-sectional design, which limits causal inferences, the use of self-reported measures of physical activity and alcohol use, potential sample bias, as the sample was predominantly non-Hispanic White and reported moderate to high socioeconomic status, and the limited generalizability of findings to other countries and populations with greater diversity (e.g., racial and ethnic minority groups, low-income, etc.).

## Conclusions

Physical activity and alcohol use were positively associated in rural cancer survivors in this study. Additional research is needed to further characterize rural cancer survivors who are physically active and drink alcohol and to identify common motivational, social, and environmental factors (e.g., stress management, peer influence, and community resources) related to these co-occurring behaviors. Comprehensive cancer control strategies are needed that address multiple health behaviors to create synergies, optimize risk reduction, and reduce health disparities.

## Data Availability Statement

The datasets analyzed for this study are available from the corresponding author upon reasonable request.

## Ethics Statement

This study was reviewed by the Institutional Review Boards at The Pennsylvania State University (STUDY00006779) and The University of Texas MD Anderson Cancer Center (Protocol ID 2021-0138), and informed consent was provided prior to participation. The patients/participants provided their written informed consent to participate in this study.

## Author Contributions

SM obtained funding, collected data, and performed statistical analyses. SM, NH, and DC designed the study. SM, HJ and DC drafted the original manuscript, and NH, HJ, and DC reviewed and edited the manuscript. All authors contributed to the article and approved the submitted version.

## Funding

This work was supported by a grant with the Pennsylvania Department of Health using Tobacco CURE Funds (TRK08-Mama-PSU-2016F to SM). SM is supported by a career development award from the National Cancer Institute at the National Institutes of Health (grant number K07 CA222335). The funders had no role in the design of the study; the collection, analysis, and interpretation of data; the writing of the manuscript; or the decision to submit the manuscript for publication. The Pennsylvania Department of Health specifically disclaims responsibility for any analyses, interpretations or conclusions.

## Conflict of Interest

The authors declare that the research was conducted in the absence of any commercial or financial relationships that could be construed as a potential conflict of interest.

## Publisher’s Note

All claims expressed in this article are solely those of the authors and do not necessarily represent those of their affiliated organizations, or those of the publisher, the editors and the reviewers. Any product that may be evaluated in this article, or claim that may be made by its manufacturer, is not guaranteed or endorsed by the publisher.

## Abbreviations

CI, Confidence interval; IPAQ, International Physical Activity Questionnaire; OR, Odds ratio; PPCC, Partnering to Prevent and Control Cancer.
